# Parenting Styles, Criticism, and Emotional Invalidation in the Parental Families of Men with Borderline Personality Disorder (BPD) and Mentally Healthy Men

**DOI:** 10.1192/j.eurpsy.2026.11906

**Published:** 2026-07-31

**Authors:** V. I. Rozhdestvenskiy, V. S. Lupashku

**Affiliations:** Department of Clinical Psychology, Saint Petersburg State Pediatric Medical University, Saint Petersburg, Russian Federation

## Abstract

**Introduction:**

Research indicates that the parental families of men with Borderline Personality Disorder **(**BPD) are often marked by high levels of criticism towards the child and emotional invalidation—a prohibition on emotional expression—which hinders the development of adaptive emotion regulation models in them. The specific contribution of each parent to the formation of this emotional climate warrants particular attention.

**Objectives:**

The aim of this study was to explore the parenting styles of mothers and fathers, and family emotional communications in the parental families of men with BPD compared to the control group of mentally healthy men.

**Methods:**

Data were collected in 2025 using an online form developed by the authors. The sample consisted of 14 men diagnosed with Borderline Personality Disorder **(**BPD) and 20 men in the control group of mentally healthy men, aged 18–25. The Family Emotional Communications Questionnaire (FEC) by Kholmogorova A.B. was used to assess criticism and emotional invalidation, and the Adolescents about Parents (ADOR) questionnaire was used to evaluate parenting styles.

**Results:**

In the parental families of men with BPD, emotional invalidation was negatively correlated with maternal Positive Interest (r = -0.59, p < 0.05) and Closeness (r = -0.76, p < 0.01), and positively correlated with maternal Hostility (r = 0.76, p < 0.01). Criticism was positively correlated with maternal Hostility (r = 0.76, p < 0.01) and negatively correlated with maternal Closeness (r = -0.66, p < 0.01). No significant correlations were found between emotional communications and paternal parenting styles in this group. In the control group, criticism was positively correlated with maternal Hostility (r = 0.58, p < 0.01) and negatively with maternal Positive Interest (r = -0.49, p < 0.05) and Closeness (r = -0.52, p < 0.05). Emotional invalidation was positively correlated with maternal Hostility (r = 0.54, p < 0.05) and negatively with maternal Positive Interest (r = -0.62, p < 0.01) and Closeness (r = -0.69, p < 0.01). Furthermore, criticism was positively correlated with paternal Inconsistency (r = 0.56, p < 0.05), Directiveness (r = 0.48, p < 0.05), and Hostility (r = 0.58, p < 0.01), and negatively correlated with paternal Closeness (r = -0.6, p < 0.01) and Positive Interest (r = -0.53, p < 0.05).

**Conclusions:**

In the parental families of men with BPD, emotional communications are closely tied to the parenting styles of mothers, while the parenting styles of fathers show no significant relationships, suggesting a lesser role of the father in shaping the family system. In the parental families of the control group, emotional communications are associated with the parenting styles of both parents, indicating a more balanced contribution from both mother and father to the family dynamics.

**Disclosure of Interest:**

None Declared

